# Exploring the Relationship Between Comorbidities and Prolonged Viral Shedding in COVID‐19: A Cycle Threshold Value‐Based Investigation

**DOI:** 10.1155/cjid/8871861

**Published:** 2026-05-12

**Authors:** Rudi Wisaksana, Patrick Philo, Floriyani Indra Putri, Chrisan Bimo Prayuda, Evan Susandi, Leonardus Widyatmoko, Bachti Alisjahbana, Iceu Dimas Kulsum, Yovita Hartantri

**Affiliations:** ^1^ Division of Tropical and Infectious Disease, Department of Internal Medicine, Faculty of Medicine Universitas Padjadjaran, Hasan Sadikin General Hospital, Bandung, Indonesia; ^2^ Department of Internal Medicine, Faculty of Medicine Universitas Padjadjaran, Hasan Sadikin General Hospital, Bandung, Indonesia; ^3^ Department of Clinical Pathology, Faculty of Medicine Universitas Padjadjaran, Hasan Sadikin General Hospital, Bandung, Indonesia; ^4^ Division of Pulmonology and Critical Illness, Department of Internal Medicine, Faculty of Medicine Universitas Padjadjaran, Hasan Sadikin General Hospital, Bandung, Indonesia

**Keywords:** autoimmune, COVID-19 infection, Ct value, HIV–AIDS, prolonged viral shedding

## Abstract

**Purpose:**

We aim to investigate which of the comorbidities is associated with prolonged viral RNA shedding among hospitalized patients with COVID‐19 illness.

**Patients and Methods:**

We conducted a retrospective cohort study of COVID‐19 patients in Hasan Sadikin General Hospital, Bandung, from March 2020 to April 2022. Data of ORF1b Ct value measurements were collected from the 1^st^ week to the 5^th^ week and presented in a line plot to see the changes in the Ct values across the course of the disease stratified to each comorbidity.

**Results:**

We included a total of 279 patients with a median age of 55‐year‐old, and male patients were the majority included. However, 68% of the original cohort was excluded due to having only a single Ct result. Based on the ORF1b Ct value data, most patients within each comorbidity group demonstrated increasing Ct values from Week 1 (*n* = 135) to Week 5 (*n* = 34). However, we found that the ORF1b Ct value in the HIV–AIDS (*n* = 12) and the autoimmune (*n* = 12) groups peaked and gradually declined. In the HIV–AIDS ORF1b, Ct values declined after 2 weeks (*n* = 2) with a statistically significant difference compared to patients without comorbidity (*n* = 65).

**Conclusion:**

This study shows that COVID‐19 patients with HIV–AIDS have prolonged viral shedding compared to patients with other comorbidities.

## 1. Introduction

Coronavirus Disease 2019 (COVID‐19) is an acute respiratory tract infection caused by severe acute respiratory syndrome coronavirus 2 (SARS‐CoV‐2) with broad clinical manifestations. Most patients have mild symptoms, but others may have more severe manifestations with acute respiratory distress, renal failure, and multiple organ dysfunction syndromes, leading to a poor prognosis [1]. Reverse transcription‐polymerase chain reaction (RT‐PCR) is currently used as the gold standard for diagnosis of COVID‐19 infection. RT‐PCR cycle threshold (Ct) value is an indirect method to measure viral load, which represents the number of amplification cycles required for the target gene to exceed a threshold level. This value is inversely related to viral load, where lower Ct values are associated with a higher number of viruses and prolonged periods of viral shedding [2].

The shedding period of SARS‐CoV‐2 begins 2‐3 days before the onset of clinical symptoms and continues for 7–14 days. A longer viral shedding period can be found in patients with severe infections [3, 4]. Resolution of the disease is characterized by a decline in RNA viral load, improvement in symptoms, rise in Ct value, and antibody titers. There are, however, several factors associated with prolonged viral shedding, including older age (> 50 years old), male sex, presence of underlying comorbidities, development of moderate/severe respiratory failure, delayed hospital admission, and high D‐dimer value at admission (> 1000 ng/mL). Preceding studies also reported that immunocompromised patients have prolonged viral shedding in contrast to immunocompetent patients, including cancer patients receiving chemotherapy, patients with autoimmune conditions receiving therapy, and HIV–AIDS patients [5–7].

Our study aimed to investigate whether these prolonged viral shedding periods are linked to the presence of comorbidities.

## 2. Materials and Methods

### 2.1. Study Design

This study is a retrospective cohort study of COVID‐19 patients in Hasan Sadikin General Hospital, Bandung, from March 2020 to April 2022, encompassed three major epidemiological phases in Indonesia: the initial waves of ancestral and Alpha variants, the Delta‐dominant surge in mid‐2021, and the early Omicron wave starting in February 2022. The inclusion criteria were age more than 18‐year‐old; confirmed COVID‐19 from nasopharyngeal swab RT‐PCR; symptomatic patients with a degree of severity from mild, moderate, to severe‐critical; and patients with either general comorbidities (hypertension, diabetes mellitus, and coronary heart disease [CHD]) or immunocompromised conditions (HIV‐AIDS, chronic kidney disease (CKD), and autoimmune disease on treatment). Patients with no or only one quantitative SARS‐CoV‐2 PCR cycle‐timed ORF1b gene results were excluded from the study. The prevalence of single‐test results was primarily due to clinical factors such as early discharge in mild cases or early mortality in critical cases and evolving national policies. During the study period, Indonesian COVID‐19 management SOPs shifted to symptom‐based discharge criteria, which no longer required follow‐up RT‐PCR for de‐isolation. Comorbidities were determined based on examination and follow‐up during hospitalization. We used the convenience sampling method in this study.

### 2.2. Variable Definition

Viral shedding persists for variable periods after symptom onset; a study of SARS‐CoV‐2 viral load and shedding kinetics reported a median duration of 16 days. In our study, with weekly follow‐up sampling, we defined prolonged viral shedding as the persistence of an ORF1b Ct value < 35 after the third week of illness [8]. COVID‐19 severity is stratified into mild, moderate, and severe‐critical and defined according to World Health Organization (WHO) guidelines [9]. Onset duration was defined as days of symptoms until hospital admission.

HIV‐AIDS is defined as patients with reactive HIV test results by the rapid chromatography antibody assay or electrochemiluminescence (ECLIA) [10]. Autoimmune is defined as patients with previously diagnosed autoimmune disease and currently receiving treatment. Chronic kidney disease (CKD) is defined according to “Kidney Disease: Improving Global Outcomes” [11]. CHD is defined according to the European Society of Cardiology (ESC) guidelines [12]. Diabetes mellitus is defined according to Indonesian Endocrinologists Association (PERKENI) guidelines [13]. Hypertension is defined as patients with systolic blood pressure above 140 mmHg and/or diastolic blood pressure above 90 mmHg in 2‐3 repeated measurements with 1–4 weeks intervals according to the 2020 International Society of Hypertension Global Hypertension Practice Guidelines [14]. Patients presenting with more than one of the above conditions were categorized exclusively into the ‘multicomorbidity’ group. Those without any history or clinical signs of the aforementioned conditions were categorized as ‘no comorbidities.’

### 2.3. Data Collection

Basic characteristics included epidemiological variables (age and sex) and clinical data (symptom onset date and illness duration, hemoglobin, leucocyte count, absolute lymphocyte count (ALC), COVID‐19 severity, and outcome), all collected at hospital admission. Ct values data were collected from the 1^st^ week to the 5^th^ week (or until the last Ct value evaluation) using the Ct gene ORF1b. Ct value data were collected and categorized into weekly intervals (Week 1–Week 5), where Week 1 was defined as the first 7 days following hospital admission.

During the study period, the protocol at Hasan Sadikin General Hospital involved periodic RT‐PCR testing, typically aimed at 7‐day intervals during the course of hospitalization. However, the exact timing and frequency of repeat testing were influenced by clinical requirements and evolving national discharge guidelines. Consequently, patients who were discharged early based on symptom resolution or those who experienced early mortality often had only a single recorded Ct value and were subsequently excluded from the longitudinal analysis.

### 2.4. Measurement

Multiple time points from admission and during hospitalization were used for testing the ORF1b gene of SARS‐CoV‐2 by RT‐PCR assays. For the measurement of the Ct value, we used semi‐quantitative methods using the LongGene thermal cycler machine (Hangzhou LongGene Scientific Ltd., China) and the mBioCoV‐19 RT‐PCR diagnostic kit (Biofarma, Indonesia) to detect the ORF1b gene and RdRP gene of SARS‐CoV‐2, using the RPP30 gene as the control. The ORF1b gene was specifically selected for this longitudinal analysis, as it represented the most complete and consistent data point across the various RT‐PCR diagnostic kits used at our institution during the study period.

### 2.5. Statistical Analysis

A frequency table was used to present baseline characteristics and demographic information. Bivariate analysis was performed to determine the relationship between clinical data and the Ct ORF1b value in the 3^rd^ week of illness using the Kruskal–Wallis test for comparative analysis and Spearman rank for correlation analysis. The test’s numerical results were further displayed with a median and interquartile range (IQR). We presented ORF1b Ct values in a line plot, divided according to the week of measurement and further stratified into groups based on comorbidities and COVID‐19 severity, to visualize changes in Ct values over the course of the disease. The normality test was performed using the Shapiro–Wilk test for continuous data and a parametric test for normally distributed data. Comparative analyses between each comorbidity group and the no‐comorbidity group were performed using independent *t*‐tests or Mann–Whitney *U* tests, depending on the data distribution. *p* value < 0.05 was considered statistically significant. To observe possible selection bias, we did sensitivity analysis comparing age, sex, severity, and outcome of patients included and excluded in the analysis. Analyses are descriptive and unadjusted for within‐patient correlation or potential confounders such as age, sex, and disease severity. Due to the retrospective nature of the secondary data and variations in data completeness across clinical variables, a formal multilevel model was not feasible. All statistical analyses were performed with IBM SPSS software version 25, Microsoft Excel 2016, and GraphPad PRISM Software Version 9.

## 3. Results

### 3.1. Baseline Characteristic

**FIGURE 1:**
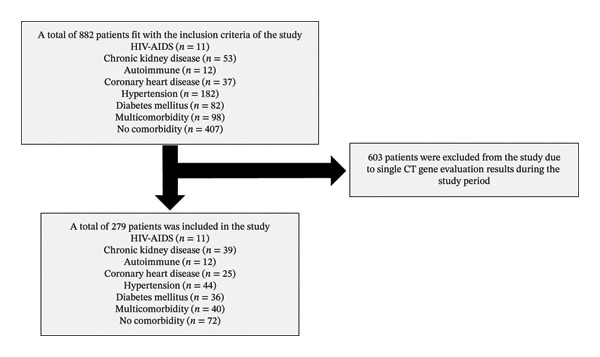
Subject inclusion flowchart.

A total of 882 patients met the study’s inclusion criteria. Among them, 603 patients were excluded due to single Ct gene evaluation results during the study period. Comparison between the included and excluded groups revealed that the excluded population had a higher mortality rate (22.3% vs. 7.2%, *p* < 0.001). Consequently, the following results are representative of a subpopulation of patients who survived the early phase of infection and remained hospitalized long enough for serial testing. Additionally, the study included 279 patients as the study subjects (Figure 1). Our sensitivity analysis did not show a difference in sex, age, or severity between the patients included in the study and those excluded. However, mortality was significantly higher in the excluded group (134/602; 22.3%) than in the included group (20/279; 7.2%; *p* < 0.001) (Supporting Table 1).

The baseline characteristics of the patients are described in Table 1. The subjects had a median age of 55 years old (IQR 42–64), and male patients were the majority, accounting for 55.2% of the subjects. Among the subjects, the group without comorbidities had the highest number of patients, with 72 individuals (25.8%), followed by the hypertension and multicomorbidity groups, with 44 patients (15.7%) and 40 patients (14.3%), respectively. Autoimmune and HIV–AIDS had the fewest number of subjects, with 12 patients (4.3%) and 11 patients (3.9%), respectively. The median Ct value of all subjects indicated an upward trend in Ct values throughout the study period, with a slight decrease observed in Week 5.

**TABLE 1:** Baseline characteristics of the study subjects.

Characteristics	Subjects (*n* = 279)
Age (median, IQR)	55 (42–64)
Sex, male (*n*, %)	154 (55.2)
Onset duration of illness (median, IQR)	4 (3–7)
Comorbidities	
No comorbidities (*n*, %)	72 (25.8)
HIV‐AIDS (*n*, %)	11 (3.9)
Chronic kidney disease (*n*, %)	39 (14.0)
Autoimmune (*n*, %)	12 (4.3)
Coronary heart disease (*n*, %)	25 (9.0)
Diabetes mellitus (*n*, %)	36 (12.9)
Hypertension (*n*, %)	44 (15.8)
Multicomorbidity (*n*, %)	40 (14.3)
Ct‐ORF1b value	
Week 1 (median, IQR)	24.9 (21.5–30.1)
Week 2 (median, IQR)	32.2 (27.3–35.1)
Week 3 (median, IQR)	35.6 (32.7–40)
Week 4 (median, IQR)	37.4 (34.1–40)
Week 5 (median, IQR)	36.8 (34.4–40)
Hemoglobin (median, IQR)	13,3 (11.4–14.6)
Leucocyte (median, IQR)	7170 (5540–10070)
Absolute lymphocyte count (ALC) (median, IQR)	1177 (888–1626.4)
O2 saturation (median, IQR)	95 (91–98)

Table 2 shows the baseline characteristics of patients in each study group. According to hemoglobin data, the lowest value was observed in patients with CKD (9.8 g/dL (IQR 7.85–10.75)), followed by HIV–AIDS patients (10.5 g/dL [IQR 9.1–13.6]), the autoimmune group (11.5 g/dL [IQR 6.1–14.2]), and the multicomorbidity group (13.2 [12.2–14.4] g/dL). The no comorbidity, hypertension, and diabetes groups tend to show normal hemoglobin distribution. Based on the leukocyte count, the HIV group exhibited the lowest value, measuring 4950 cells/μL (IQR 4220–9650), followed by the autoimmune group with a leukocyte count of 5700 cells/μL (IQR 4735–9180). Furthermore, the HIV group also displayed the lowest ALC of 828 cells/μL (IQR 200–1430), with a significant difference compared to the no comorbidities group (1259.1 cells/μL [IQR 994.4–1667.8]). The CKD group also showed the second lowest ALC with a median of 1020 (IQR 590–1666) cells/μL. In terms of COVID severity, HIV‐AIDS patients had the highest proportion of mild COVID cases (45.5%), CKD patients had the highest proportion of moderate COVID cases (56.4%), and DM patients had the highest proportion of severe COVID cases (44.4%). The outcome data revealed that patients with DM had the highest mortality rate, followed by patients with CKD, with 6 and 5 patients, respectively. Due to the distinctive ALC among the HIV, autoimmune and CKD patients, we explored the correlation between ORF1b Ct values and ALC. This analysis did not show a significant correlation (Spearman’s *ρ* = 0.034 (−0.11–0.17), *p* = 0.62).

**TABLE 2:** Baseline characteristics of the study subjects stratified by comorbidity.

Variables	No comorbidities (*n* = 72)	HIV–AIDS (*n* = 11)	Autoimmune (*n* = 12)	Chronic kidney disease (*n* = 39)	Coronary heart disease (*n* = 25)	Diabetes mellitus (*n* = 36)	Hypertension (*n* = 44)	Multicomorbidity (*n* = 40)
Age (median, IQR)	45.5 (35–56.8)	35 (27–37)^∗∗^	39 (30.3–54.8)	49 (40–67)^∗^	58 (51.5–66.5)^∗∗^	58 (48.3–65)^∗∗^	59.5 (50.1–64.8)^∗∗^	60.5 (54–67)^∗∗^
Sex, male (*n*, %)	40 (55.6)	7 (63.6)	3 (25)	22 (56.4)	15 (60)	22 (61.1)	28 (63.6)	17 (42.5)
Onset duration (median, IQR)	5 (3–7)	4 (3–14)	4 (3–6)	3 (2–7)	6 (3.25–8.8)	4 (2–7)	4 (3–7)	4 (3–6)
Ct‐ORF1b value								
Week 1 (median, IQR)	26.3 (21.6–30)	23.5 (21.3–23.7)	24.1 (22.1–29.6)	24.4 (21.3–28)	23.7 (21.8–28)	27.7 (21.3–31.7)	26.7 (21.5–31)	23.7 (21.6–28.8)
Week 2 (median, IQR)	32 (27.5–34.6)	27.7 (19.6–36.6)	33.7 (28.2–34.1)	33.5 (28.9–37.4)	32.3 (28.2–36.3)	32.2 (27.9–35.1)	32.4 (27.6–34.3)	30.6 (26.1–34.2)
Week 3 (median, IQR)	35.8 (33.5–38.1)	23.9 (20.4–29.6)^∗^	40 (36–40)	35.4 (32.8–40)	36.6 (35.4–40)	35.4 (32.7–40)	33.9 (30.9 = 39.3)	35.9 (33.8–40)
Week 4 (median, IQR)	40 (35.3–40)	23.7 (19.3–30.8)^∗∗^	32.1	37 (36.3–38)	40 (34.9–40)	34.4 (30.5–35.9)^∗∗^	37.5 (33.5–40)	34.8 (31.2–40)^∗^
Week 5 (median, IQR)	40 (37.7–40)	19.3 (18.7–20)^∗^	28.6	35.3 (33.7–37.7)	37.2 (34.1–40)	38.6 (34.5–40)	36.1 (34.4–40)	37.2 (34.3–40)
Hemoglobin (median, IQR)	13.6 (12.6–14.7)	10.5 (9.1–13.6)^∗^	11.5 (6.1–14.2)^∗^	9.8 (7.85–10.75)^∗∗^	13.6 (12.7–14.8)	14.1 (12.9–14.9)	14.3 (13–15.4)^∗^	13.2 (12.2–14.4)
Leucocyte (median, IQR)	7070 (5562.5–9240)	4950 (4220–9650)	5700 (4735–9180)	7650 (5640–11710)	8280 (7080–10140)^∗^	7365 (5705–11417.5)	6965 (5175–10322.5)	7169.5 (5572.5–8557.5)
Absolute lymphocyte count (median, IQR)	1259.1 (994.4–1667.8)	828 (200–1430)^∗∗^	1275.1 (785.9–1473.3)	1020 (590–1666)	1116 (872.4–1456.2)	1180.6 (942.8–1684.7)	1370.1 (941–1771.5)	1108.4 (899.6–1500.5)
O2 saturation (median, IQR)	96 (93–98)	98 (94.5–98)	96.5 (96–98.5)	93.5 (89–97)	94 (85.5–97.5)	94 (89.3–97)	96 (91–98)	94 (88–97)^∗^
COVID‐19 severity								
Mild COVID‐19 (*n*, %)	22 (30.6)	5 (45.5)	4 (33.3)	5 (12.8)	8 (32)	5 (13.9)	8 (18.2)	5 (12.5)
Moderate COVID‐19 (*n*, %)	35 (48.6)	6 (54.5)	5 (41.7)	22 (56.4)	11 (44)	15 (41.7)	23 (52.3)	21 (52.5)
Severe‐critical COVID‐19 (*n*, %)	15 (20.8)	0	3 (25)	12 (30.8)	6 (24)	16 (44.4)	13 (29.5)	14 (35)
Outcome								
Death (*n*, %)	2 (2.8)	0	2 (16.7)	5 (12.8)	1 (4)	6 (16.7)	2 (4.5)	2 (5)
Alive (*n*, %)	70 (97.2)	11 (100)	10 (83.3)	34 (87.2)	24 (96)	30 (83.3)	42 (95.5)	38 (95)

^∗^ *p* value < 0.05 compared to patient with no comorbidity.

^∗∗^ *p* value < 0.001 compared to patient with no comorbidity.

### 3.2. Ct ORF1b Line Plot

**FIGURE 2:**
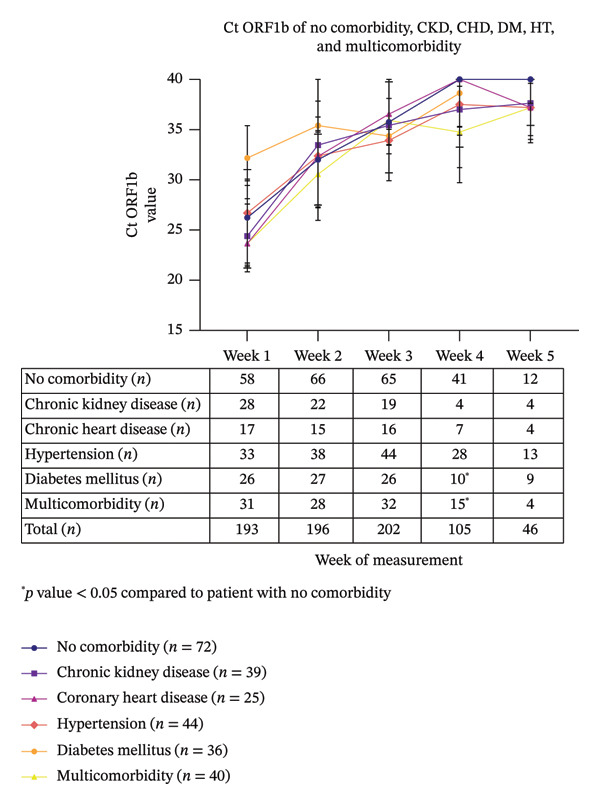
Line plot graphic comparison between no comorbidity, chronic kidney disease, coronary heart disease, hypertension, diabetes mellitus, and multicomorbidity patients ^∗^ *p* value < 0.05 compared to patient with no comorbidity (diabetes vs. no comorbidity in Week 4 *p* value: 0.0162).

**FIGURE 3:**
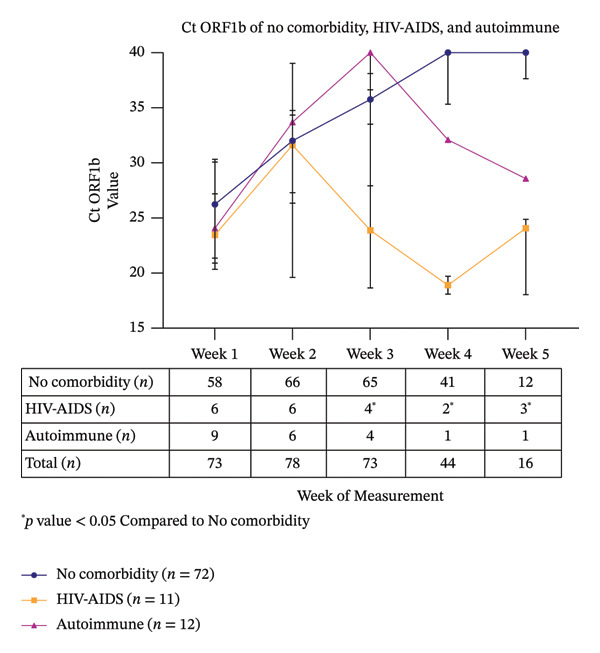
Line plot graphic comparison between no comorbidity, HIV–AIDS, and autoimmune patients ^∗^ *p* value < 0.05 compared to no comorbidity (HIV–AIDS vs. no comorbidity *p* value: 0.0053 [Week 3], < 0.0001 [Week 4], and < 0.0001 [Week 5]).

We created the line plots to observe the Ct ORF1b trend over the course of 5 weeks. We divided the plots into 2 graphs, categorized by comorbidities and the severity of COVID‐19. Based on the Ct ORF1b stratified by comorbidities, most patients demonstrated an increasing trend (Figure 2). However, in the HIV–AIDS group, the Ct ORF1b value peaked in Week 2 and was notably lower than the no‐comorbidity group at Week 3 (*n* = 4). While a significant declining trend was observed in the few remaining patients at week 4 (*n* = 2) and Week 5 (*n* = 3) (Figure 3). Similarly, in the autoimmune patient, a decline was noted after Week 3 (*n* = 4), but data for weeks 4 and 5 represent a single illustrative case. Statistically significant differences were observed in Ct ORF1b results on Week 4 for diabetes mellitus and multicomorbidity groups, compared to the no‐comorbidity group. In terms of COVID severity, we did not find any significant difference in the value and kinetics of the Ct ORF1b figures (Supporting Figure 1).

## 4. Discussion

We have observed the difference in Ct value changes across the course of the disease in COVID‐19 patients with different comorbidities. We observed that HIV–AIDS and autoimmune patients tended to have lower Ct ORF1b values starting at Week 3. While the data suggests a declining trend thereafter, the small number of patients remaining in these cohorts at Weeks 4 and 5 is a result of clinical outcomes and shifting discharge policies, limiting definitive conclusions regarding long‐term kinetics in these subpopulations.

The median duration of SARS‐CoV‐2 RNA shedding was 23 days, with shedding beginning 2‐3 days before the onset of symptoms and continuing for 7–14 days; however, in severe infections, the shedding could persist for 20–31 days [3, 4]. Viral loads are used to represent the infectious viral shedding with higher viral loads related to prolonged viral shedding. Studies have reported that immunocompromised patients have prolonged viral shedding in contrast to immunocompetent patients, including patients with autoimmune conditions receiving therapy and HIV–AIDS [6, 7]. It is critical to distinguish between the detection of viral RNA and the presence of infectious virus. Our study measures the kinetics of viral RNA fragments; however, a low Ct value, while a useful proxy, does not definitively confirm the presence of viable, culturable SARS‐CoV‐2. However, prolonged viral shedding might be related to prolonged viral transmission, which leads to prolonged hospitalization and isolation. Previous study also reported that the prolonged shedding period is associated with in‐hospital delirium and increased 6‐month mortality [15]. Patients with prolonged viral shedding have a higher risk of developing long‐COVID syndrome, characterized by persistent symptoms of COVID‐19, which is highly associated with higher morbidity and several psychiatric symptoms, including depression, anxiety, post‐traumatic distress, cognitive impairment, and sleeping disturbance [16, 17].

Preceding reports demonstrated that prolonged viral shedding (85 days) in HIV–AIDS patients may occur without the development of fatal pneumonia. The protracted course of viral shedding and the absence of severe COVID‐19 might be related to lymphopenia and reduced CD4 T‐cell count in this patient [18]. Infection by HIV is characterized by impairment of the adaptive immune system, with destruction of CD4+ T‐cells, an increase in CD8+ T‐cells, activation and exhaustion of T‐cells, reduced activity of T follicular helper (Tfh) cells, and dysfunction and abnormal activation of B‐cells. This immunodeficiency nature of HIV–AIDS might reduce antibody response and hinder viral clearance, resulting in an extended disease course and prolonged viral shedding [19, 20]. Conversely, long‐term immunosuppression might lower the severity of the immune system reaction and prevent the occurrence of cytokine storms commonly associated with severe COVID‐19 infection. These contribute to a prolonged course of disease, albeit with milder symptoms, lower morbidity, and reduced mortality rates [21, 22]. Our study has also observed prolonged viral shedding in HIV–AIDS patients with the absence of severe COVID‐19 infection and no mortality cases.

Our observation also found a COVID‐19 patient with an autoimmune disease also has prolonged viral shedding. Our observation of prolonged shedding in the autoimmune group, specifically the sustained low Ct values in one patient through Week 5, serves as an illustrative case of how systemic autoimmunity and immunosuppressive therapy might interfere with viral clearance. However, given the rarity of these cases in our cohort, these findings require validation in larger, dedicated autoimmune‐COVID‐19 studies. A previous study using the COVID‐19 Global Rheumatology Alliance (GRA) Vaccine Survey demonstrated that 24.2% of patients with a systemic autoimmune rheumatic disease (SARD) have met the CDC definition for prolonged COVID‐19 symptoms (≥ 28 days), which might be associated to COVID‐19 severity, older age, and comorbidities. Other factors that might be related to prolonged shedding include altered immunity with the use of immunosuppressive medications including the use of glucocorticoids and disease‐modifying antirheumatic drugs [21, 23]. Glucocorticoids continue to be the preferred option for treating inflammation and suppressing the immune system by causing apoptosis in developing thymocytes and T cells. Additionally, it effectively reduces the production of cytokines in T cells and decreases the number of circulating B cells [24]. Several immunosuppressive drugs like cyclosporine, tacrolimus, mycophenolate mofetil, and azathioprine induce immunosuppression by causing inhibition in B cell and T cell proliferation [25].

CKD is characterized by significant innate and adaptive immune dysfunction. While previous research suggests CKD patients experience prolonged COVID‐19 viral shedding compared to those without renal impairment, our study found no such difference. Uremia typically depletes naïve T cells via reduced thymic output and apoptosis, while simultaneously increasing B‐cell apoptosis through downregulated Bcl‐2 expression. The discrepancy in our findings may stem from the limited sample size of CKD patients tested at Weeks 4 and 5 (*n* = 4/28) [26, 27].

We observed no significant differences in Ct value trends among patients with CHD, hypertension, or diabetes mellitus compared to those without comorbidities. Conversely, existing literature indicates these populations face a higher risk of COVID‐19 cytokine storms despite similar viral loads [28]. In CHD, CD4+ T‐cells drive atherosclerosis via proinflammatory cytokines (IFN‐γ, IL‐2, IL‐3, TNF‐α, and TNF‐β) that amplify immune recruitment and antibody production [29, 30]. Hypertension is similarly marked by immunosenescence, featuring overactive CD8+ cells, increased cytokine‐secreting B cells, NK cells, and M‐1 macrophages, alongside reduced anti‐inflammatory Treg cells [31, 32]. In Type 2 Diabetes (T2DM), T‐cell overactivation and skewed Th1/Treg ratios promote inflammatory progression; furthermore, hyperglycemia facilitates SARS‐CoV‐2 replication and ACE2 expression in pulmonary monocytes, triggering reactive oxygen species (ROS) and systemic cytokine release [33, 34]. These combined mechanisms frequently culminate in a cytokine storm.

Overactivation of T cells and inflammatory pathways play an important role in the progression of T2DM, which is characterized by the increase of proinflammatory Th1 cells, the decrease of anti‐inflammatory Treg cells, and the increase of CD8^+^ T cells and B cells. The hyperglycemic state of T2DM can facilitate SARS‐CoV‐2 replication and ACE2 expression in monocytes accumulated in the lungs of COVID‐19 patients, inducing ROS production and cytokine over‐release. These conditions altogether can lead to cytokine storm [33, 34].

Our study has several limitations. First, we did not design the study from the beginning by allocating serial PCR testing for all of the patients. Therefore, we found a high proportion of patients who only have one PCR test due to early mortality or were discharged after a single test was excluded from this study. This situation may bias our observation to the more severe patient who is hospitalized for a longer term. This was compounded by changes in local health regulations in Indonesia that moved away from test‐based discharge. Therefore, our results regarding prolonged viral shedding may be skewed toward patients with more stable or protracted clinical courses and may not be generalizable to those with rapid, fatal disease trajectories. Second, we have limited data, especially on the fourth and fifth week of illness. Similarly, the data we have may be biased towards the more severe patient who was still being indicated for hospitalization. The potential for confounding by age and disease severity must be considered when interpreting these findings. In our cohort, patients in the diabetes, hypertension, and multicomorbidity groups were generally older than those in the HIV–AIDS group. Advanced age is closely linked to immunosenescence in elderly which can independently prolong viral clearance regardless of specific comorbidities. Similarly, baseline COVID‐19 severity influences viral load kinetics [35, 36]. However, because severity often dictates the frequency of testing in a clinical setting which may introduce detection bias. Third, our study did not adjust for the specific SARS‐CoV‐2 variant or individual vaccination status. Vaccination has been shown to reduce the duration of infectious shedding, which might lead to an underestimation of shedding duration in our later subcohorts compared to patients from 2020. We realized this limitation; therefore, our aim is to highlight the most prominent comorbidities in relation to prolonged viral shedding. In this study, we analyzed only the ORF1b gene target, although other gene targets may also provide important indicators of viral load. This restriction may limit the generalizability of our findings to other assays or gene targets. However, the longitudinal Ct trajectories observed in this study must be interpreted within the context of evolving viral variants and medical interventions. The emergence of the Delta variant in 2021 was associated with higher peak viral loads and potentially longer shedding compared to ancestral strains [37]. It is also important to consider the role of therapeutics; while corticosteroids are life‐saving in severe COVID‐19, some evidence suggests they may paradoxically delay viral RNA clearance. Because patients with diabetes, hypertension, and multicomorbidity were more likely to receive these treatments due to disease severity (perpendek).

These findings highlight the need for refined patient management strategies. Ct values above 30–35 typically indicate nonviable virus and low transmission risk, while Ct < 35 remains a reliable marker of infectiousness [38]. Prolonged viral shedding in immunocompromised patients increases nosocomial transmission risk, particularly in close‐contact settings [39]. Extended isolation, however, contributes to substantial psychological distress, underscoring the need for integrated mental health support [40, 41]. Clinically, balancing infection control with immune restoration and complication management is challenging. Given the limitations of Ct values alone, isolation decisions should be individualized, integrating Ct thresholds with clinical status, symptom duration, immune function, expert input, and, when feasible, viral culture or genomic sequencing.

## 5. Conclusion

This study aims to observe the difference in Ct value changes across the course of the disease in COVID‐19 patients with different comorbidities. We observed prolonged viral shedding in COVID‐19 patients with HIV–AIDS characterized by a slower decline in Ct values through the third week of illness. Patients with other comorbidities (diabetes, hypertension, and multiple comorbidities) did not show longer viral shedding duration. Additional studies focused on the risk factors of prolonged viral shedding utilizing longitudinal modeling and adjustment with more factors to confirm these independent associations and its linkage to pathophysiology of COVID‐19 severity, management strategy, and outcome are needed.

**Nomenclature**
COVID‐19: Coronavirus Disease 2019
SARS‐CoV‐2: Severe acute respiratory syndrome coronavirus 2
RT‐PCR: Reverse transcription‐polymerase chain reaction
CT: Cycle threshold
WHO: World Health Organization
ECLIA: Electrochemiluminescence
CKD: Chronic kidney disease
CHD: Coronary heart disease
ESC: European Society of Cardiology
ALC: Absolute lymphocyte count
IQR: Interquartile range
Tfh: T follicular cell
GRA: Global Rheumatology Alliance
SARD: Systemic autoimmune rheumatic disease
ROS: Reactive oxygen species

## Funding

No funding was received for this manuscript.

## Ethics Statement

This study was approved by the Ethics Committee or Institutional Review Board of Hasan Sadikin General Hospital with ethics number LB.02.01/*X*.6.5/100/2020, 6 May 2020 and was conducted in accordance with the Declaration of Helsinki. Written informed consent was waived by the Ethics Committee because of the secondary use of medical data. All data were kept anonymous.

## Conflicts of Interest

The authors declare no conflicts of interest.

## Supporting Information

Additional supporting information can be found online in the Supporting Information section.

## Supporting information


**Supporting Information** Supporting Table 1. Sensitivity analysis between groups included and excluded in the analysis. Supporting Figure 1. Line plot graphic comparison between mild, moderate, and severe COVID‐19 patients.

## Data Availability

The data that support the findings of this study are available from the corresponding author upon reasonable request.
