# Supplementary material for: Exploring the Relationship Between Comorbidities and Prolonged Viral Shedding in COVID‐19: A Cycle Threshold Value‐Based Investigation
Source: Can J Infect Dis Med Microbiol. 2026 May 12;2026:8871861. doi: 10.1155/cjid/8871861 (PMC13161999; doi:10.1155/cjid/8871861)
Supplement: Supplementary file 1 — Supporting Information Supporting Table 1. Sensitivity analysis between groups included and excluded in the analysis. Supporting Figure 1. Line plot graphic comparison between mild, moderate, and severe COVID‐19 patients. [file CJID-2026-8871861-s001.zip › Supplementary Figure 1.docx]

**Supplementary Figure 1. Line plot graphic comparison between mild, moderate, and severe COVID-19 patients**
