# Supplementary material for: Exploring the Relationship Between Comorbidities and Prolonged Viral Shedding in COVID‐19: A Cycle Threshold Value‐Based Investigation
Source: Can J Infect Dis Med Microbiol. 2026 May 12;2026:8871861. doi: 10.1155/cjid/8871861 (PMC13161999; doi:10.1155/cjid/8871861)
Supplement: Supplementary file 1 — Supporting Information Supporting Table 1. Sensitivity analysis between groups included and excluded in the analysis. Supporting Figure 1. Line plot graphic comparison between mild, moderate, and severe COVID‐19 patients. [file CJID-2026-8871861-s001.zip › Supplementary File_1.docx]

| **Variables** | **Total** | **Included**  **N=279** | **Excluded**  **N=603** | **p-value** |
| --- | --- | --- | --- | --- |
| **Age (median, IQR)** | 54 (42-64) | 55 (42-64) | 54 (41-65) | 0.879 |
| **Sex (n,%)** |  |  |  |  |
| Female | 426 | 125 (44.8%) | 301 (49.9%) | 0.158 |
| Male | 456 | 154 (55.2%) | 302 (50.1%) |  |
| **Severity** **(n,%)** |  |  |  |  |
| Mild | 172 | 62 (22.2%) | 110 (18.3%) | 0.75 |
| Moderate | 414 | 138 (49.5%) | 276 (45.9%) |  |
| Severe | 294 | 79 (28.3%) | 215 (35.8%) |  |
| **Outcome (n,%)** |  |  |  |  |
| Death | 154 | 20 (7.2%) | 134 (22.3%) | 0.0001** |
| Alive | 727 | 259 (92.8%) | 468 (77.7%) |  |

**Supplementary Table 1. Sensitivity analysis between groups included and excluded in the analysis**

**p-value <0.001

**Supplementary Figure 1. Line plot graphic comparison between mild, moderate, and severe COVID-19 patients**
